# Area of the IBD Disk Correlated Strongly with Disease Activity Compared with the Conventionally Used IBD Disk Score

**DOI:** 10.1093/ecco-jcc/jjab138

**Published:** 2021-07-30

**Authors:** Péter Bacsur, Klaudia Farkas, Tamas Molnar

**Affiliations:** Department of Medicine, University of Szeged Faculty of Medicine, Szeged, Hungary

We read with great interest the recently published article by Le Berre *et al*. assessing the validity of the Inflammatory Bowel Disease [IBD] Disk, a self-administered instrument to measure IBD-related disability.^1^ It is well known that IBD has a significant burden on all areas of the patients’ lives. Quick, simple, and effective questionnaires are needed to reflect the subjective symptoms and functional conditions of the patients both for themselves and for the physicians at regular visits. The Inflammatory Bowel Diseases Disability Index [IBD-DI] is effective, but its daily use is limited by the length of the questionnaire.^2,3^ The VALIDation study verified the usability of the IBD Disk, a simple, visual tool for measuring disability along 10 axes, filled by the patients. This elegant study demonstrated the significant correlations of the IBD Disk score with clinical factors such as gender, physician global assessment, Harvey‐Bradshaw Index, and pMayo scores, over and above scores intended to be associated with C-reactive protein [CRP] and fecal calprotectin.

Size of the IBD Disk area indicates the severity of the IBD-related disability of the patients, so we hypothesised that the area of the polygon would correlate with clinical factors better than the sum of the item scores. Therefore, we compared the correlation of the clinical characteristics and disease activities of IBD with the area of the IBD Disk polygon and with the sum of the item scores.

In this study, 45 consecutive IBD patients [male/female ratio 21/24, average age 42 years], treated with infliximab (25 Crohn’s disease [CD], 16 ulcerative colitis [UC]) or with vedolizumab [three CD, one UC] at our tertiary centre, were enrolled; 20 % of patients suffered from perianal manifestations. The average disease duration time was more than 13 years. We calculated pMayo and Crohn’s Disease Activity Index [CDAI] scores, measured serum CRP, haematocrit, haemoglobin, leukocyte, thrombocyte, iron, and calprotectin levels, and asked the patients to fill IBD Disk questionnaire every second month throughout the 12-month follow-up period. In this way we assessed linear regressions of the connections used in all the four visits’ data per model. Our results showed that CDAI and pMayo scores associated significantly with IBD Disk area [CDAI R^2^ = 0.05, pMayo R^2^ = 0.18] as well as with the sum of the item scores [CDAI R^2^ = 0.07, pMayo R^2^ = 0.07]. Stronger correlation was shown between the IBD Disk area and haematocrit [R^2^ = 0.071] and haemoglobin [R^2^ = 0.08] than using the sum of the scores [haematocrit R^2^ = 0.055, haemoglobin R^2^ = 0.06]. None of the other examined biochemical parameters correlated with the IBD Disk scores.

The COVID-19 pandemic highlighted the importance of telemedical instruments during long-term follow-up among IBD patients. Our study demonstrates that using the area of the IBD Disk polygon could help to apply a treat-to-target approach during the visits more accurately.

## References

[1] Le Berre C , Flamant M, Bouguen G, et al VALIDation of the IBD-Disk instrument for assessing disability in inflammatory bowel diseases in a French cohort: the VALIDate study. J Crohns Colitis 2020;14:1512–23. 32417910 10.1093/ecco-jcc/jjaa100

[2] Peyrin-Biroulet L , Cieza A, Sandborn WJ, et al; International Programme to Develop New Indexes for Crohn’s Disease [IPNIC] group. Development of the first disability index for inflammatory bowel disease based on the international classification of functioning, disability and health. Gut 2012;61:241–7. 21646246 10.1136/gutjnl-2011-300049 PMC3245899

[3] Ghosh S , Louis E, Beaugerie L, et al Development of the IBD disk: a visual self-administered tool for assessing disability in inflammatory bowel diseases. Inflamm Bowel Dis 2017;23:333–40. 28146002 10.1097/MIB.0000000000001033 PMC5319390

